# Diagnosis and Management of Iatrogenic Hemobilia Secondary to Transjugular Intrahepatic Portosystemic Shunt Procedure

**DOI:** 10.7759/cureus.7629

**Published:** 2020-04-10

**Authors:** Gino Puntel, Giovanni Puppini, Simone Perandini, Riccardo De Robertis, Stefania Montemezzi

**Affiliations:** 1 Radiology, Azienda Ospedaliera Universitaria Integrata Verona, Verona, ITA

**Keywords:** hepatology, interventional radiology, liver, transjugular intrahepatic portosystemic shunt, hemobilia, bleeding, complication, iatrogenic, endovascular, embolization

## Abstract

A patient with end-stage liver disease and subsequent refractory ascites was treated with a transjugular intrahepatic portosystemic shunt (TIPS) procedure. The procedure was complicated by massive gastrointestinal hemorrhage with associated rettorragia. Computed tomography angiography (CT-A) was performed and revealed haemobilia due to an artero-biliary fistula between the right hepatic artery and an intrahepatic biliary branch. The patient underwent an attempt at percutaneous embolization. Bleeding was successfully stopped by the embolization of the fistula with coils.

Hemobilia is a rare cause of upper gastrointestinal bleeding with an increasing incidence due to the widespread use of invasive hepatobiliary procedures. Hemobilia is an uncommon complication of TIPS procedures. Nowadays, transcatheter embolization is the gold standard in the management of hemobilia.

## Introduction

Hemobilia occurs when a fistulous track forms between a hepatic vessel (hepatic artery or portal vein) and the intrahepatic biliary system or choledocus. The most frequent causes are iatrogenic after hepatobiliary system procedures and trauma [1-2]. The treatment of hemobilia is aimed at stopping the bleeding without interfering with the normal anatomy of the biliary and vascular structures. We report iatrogenic hemobilia as a complication of the transjugular intrahepatic portosystemic shunt (TIPS) procedure, which was successfully managed by transcatheter embolization [3].

## Case presentation

**Figure 1:**
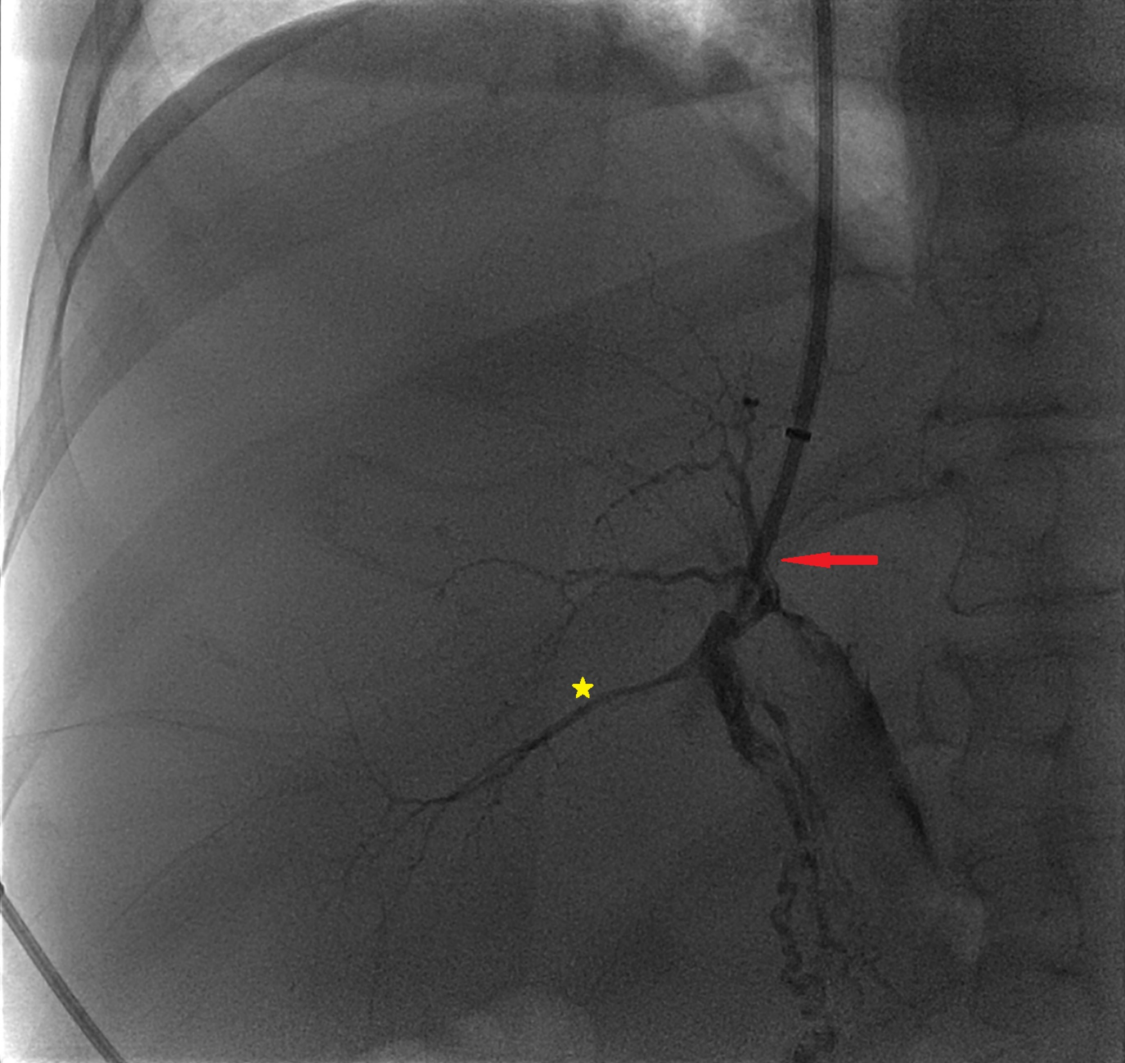
Intraprocedural fluoroscopy Right hepatic artery branches' opacification (yellow star) was observed during an attempt to position the Colapinto needle (red arrow).

A 59-year-old man with alcohol abuse-related liver cirrhosis was treated for refractory ascites (model end-stage liver disease (MELD) score 17) by means of a transjugular intrahepatic portosystemic shunt (TIPS) procedure. The procedure succeeded uneventfully, however, transient right hepatic artery branches opacification was observed during an attempt to position the Colapinto needle within the portal venous tree (Figure 1).

**Figure 2:**
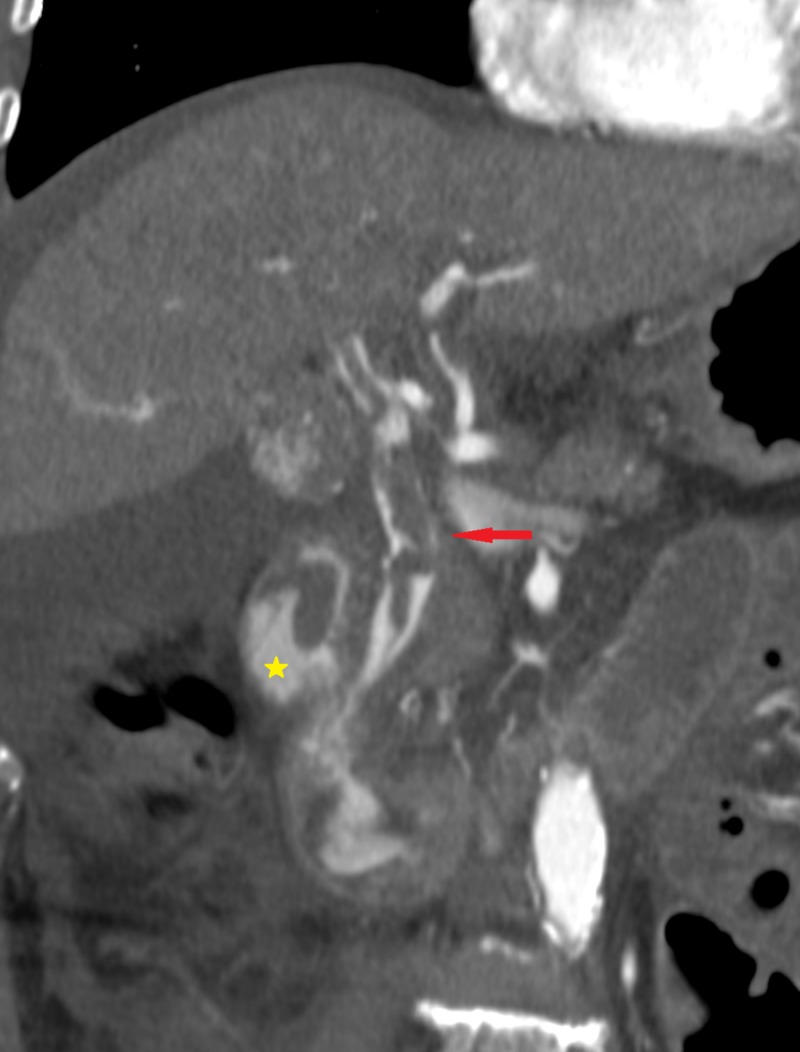
CT angiography Arterial phase images revealed contrast media filling of the biliary tree, the choledocus (red arrow), and the duodenum (yellow star).

The patient was discharged from the operating room with no signs of complications. Two days later, a massive gastrointestinal hemorrhage with associated rettorragia occurred, leading to a significant decrease in hemoglobin (from 12 mg/dl to 6 mg/dl); concomitant increased total bilirubin blood levels was detected (from 10 µmo/L to 290 µmo/L). Computed tomography angiography (CT-A) was performed to assess haemobilia. Arterial phase images revealed contrast media filling of the biliary tree, the choledocus and the duodenum (Figure 2).

**Figure 3:**
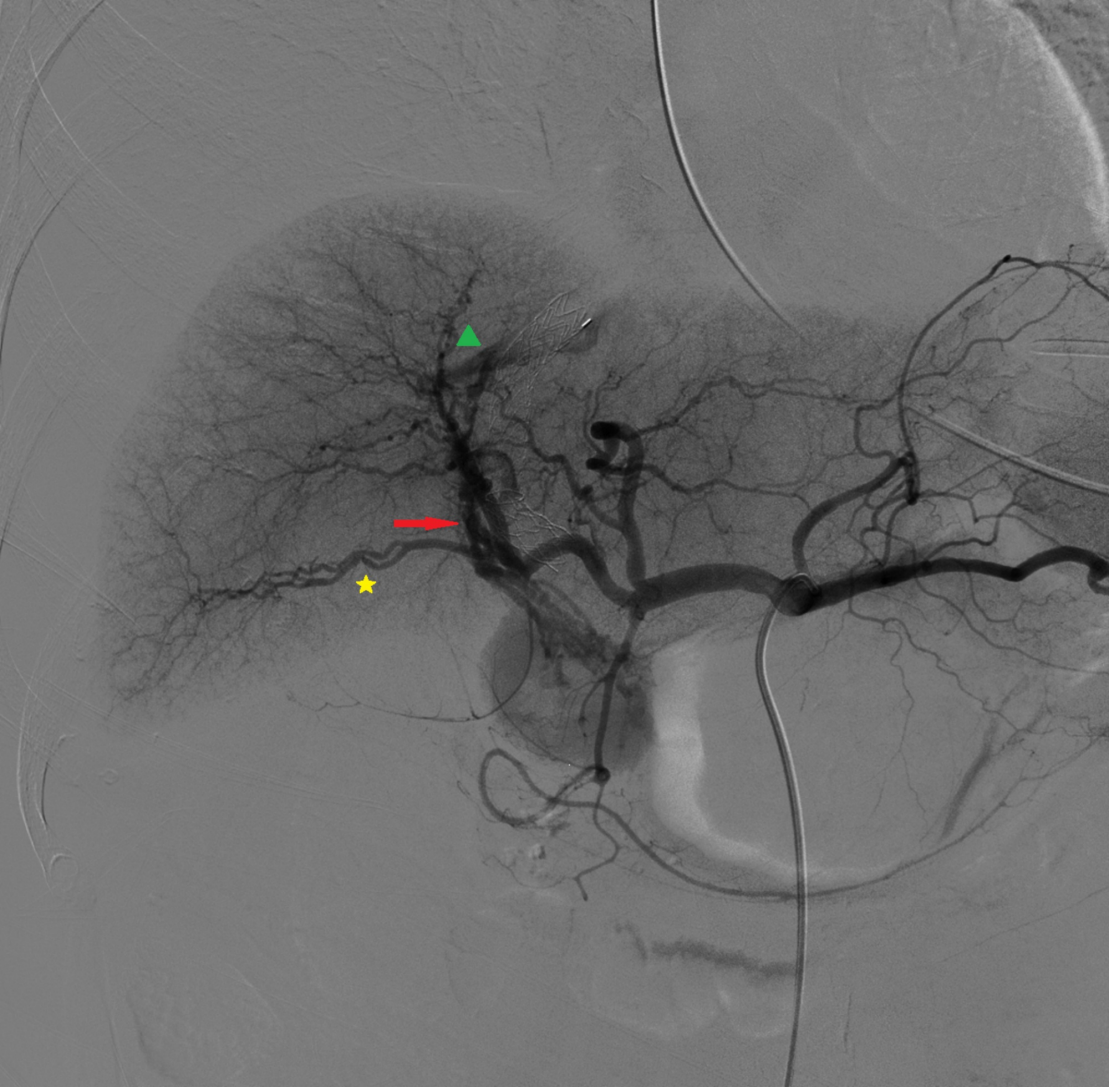
Hepatic artery angiography, early phase Fistula (red arrow) between the proximal branch of the right hepatic artery (yellow star) and the right hepatic vein (green triangle)

**Figure 4:**
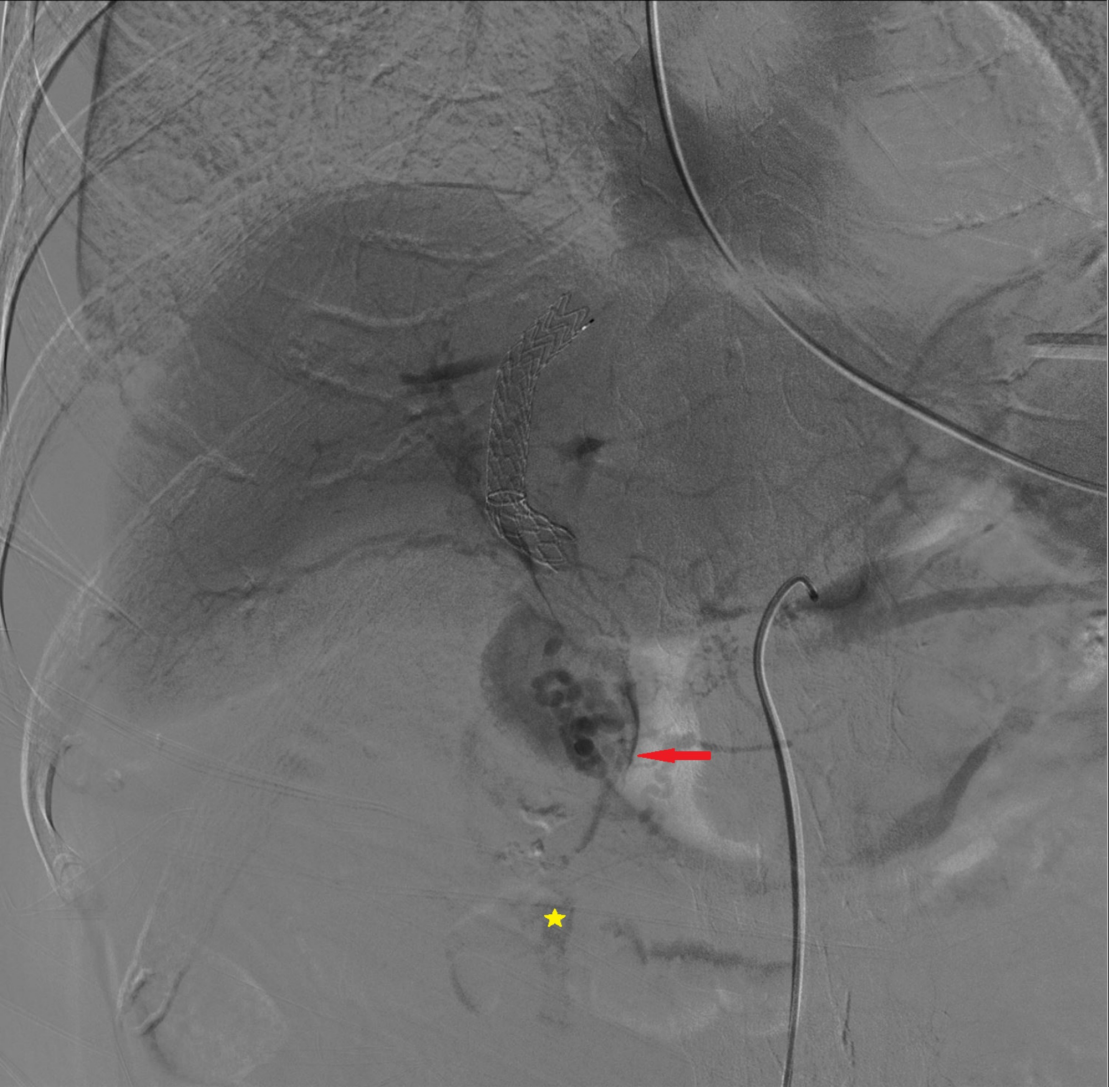
Hepatic artery angiography, late phase Delayed opacification of the biliary tree (red arrow) and the duodenum (yellow star)

The patient underwent an attempt at percutaneous embolization. Selective angiography of the celiac trunk revealed an arteriovenous-biliary fistula between the proximal branch of the right hepatic artery, the right hepatic vein, and the right biliary system (Figure 3 and Figure 4).

**Figure 5:**
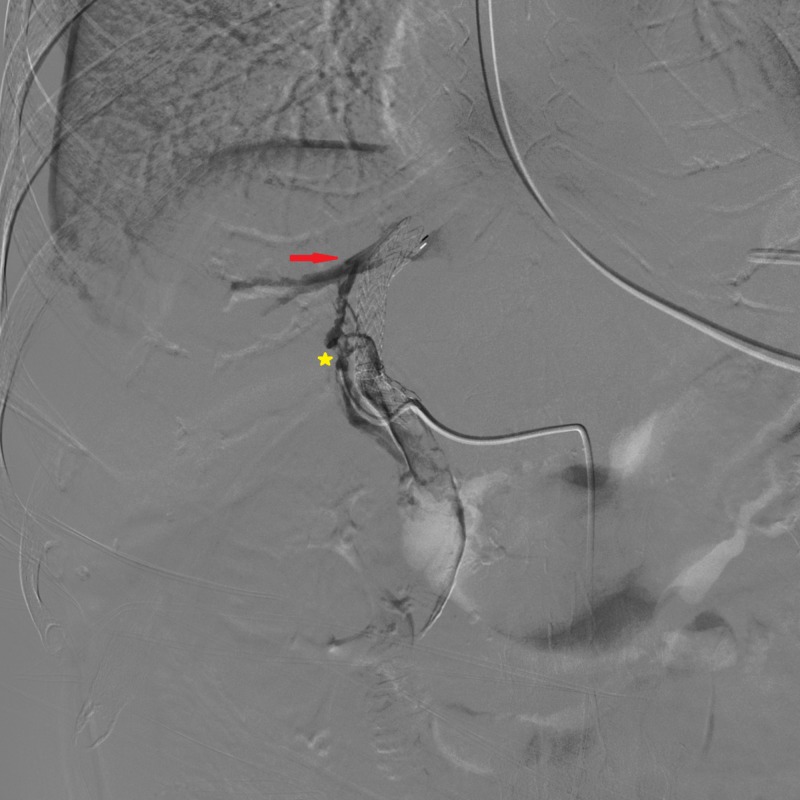
Angiography from a microcatheter within the fistula, early phase Opacification of the hepatic vein (red arrow) and the biliary tree (yellow star)

**Figure 6:**
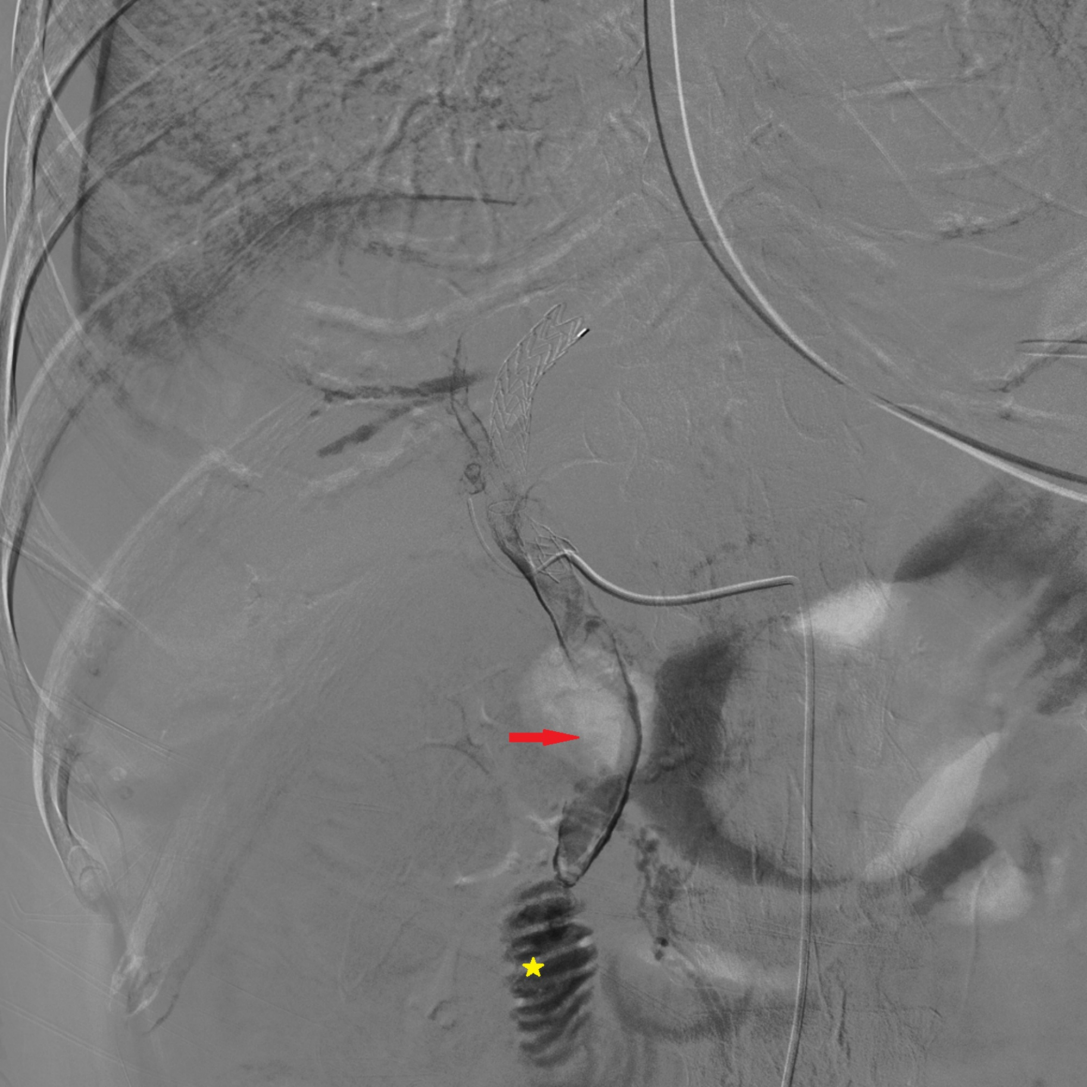
Angiography from the microcatheter within the fistula, late phase Opacification of the choledocus (red arrow) and the duodenum (yellow star)

A microcatheter (Progreat 2.7 F; Terumo Corporation, Tokyo, Japan) was advanced within the fistula; contrast media injection confirmed opacification of the hepatic vein, the biliary tree, the choledocus and the duodenum (Figure 5 and Figure 6).

**Figure 7:**
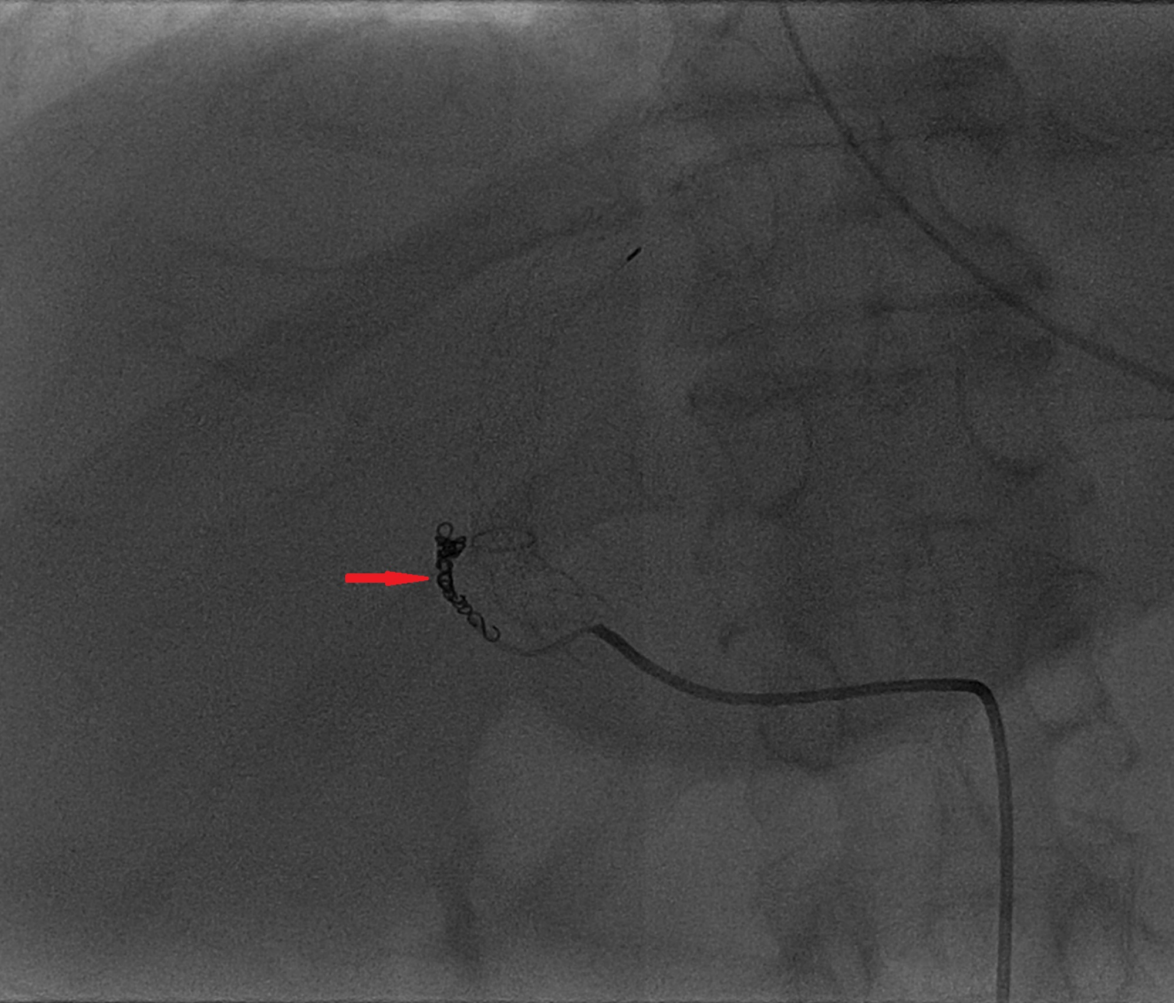
Pushable microcoils (red arrow) inside the fistulous tract

Pushable microcoils (0.018 Coils; Boston Scientific, Marlborough, Massachusetts) were released inside the fistulous tract (Figure 7).

**Figure 8:**
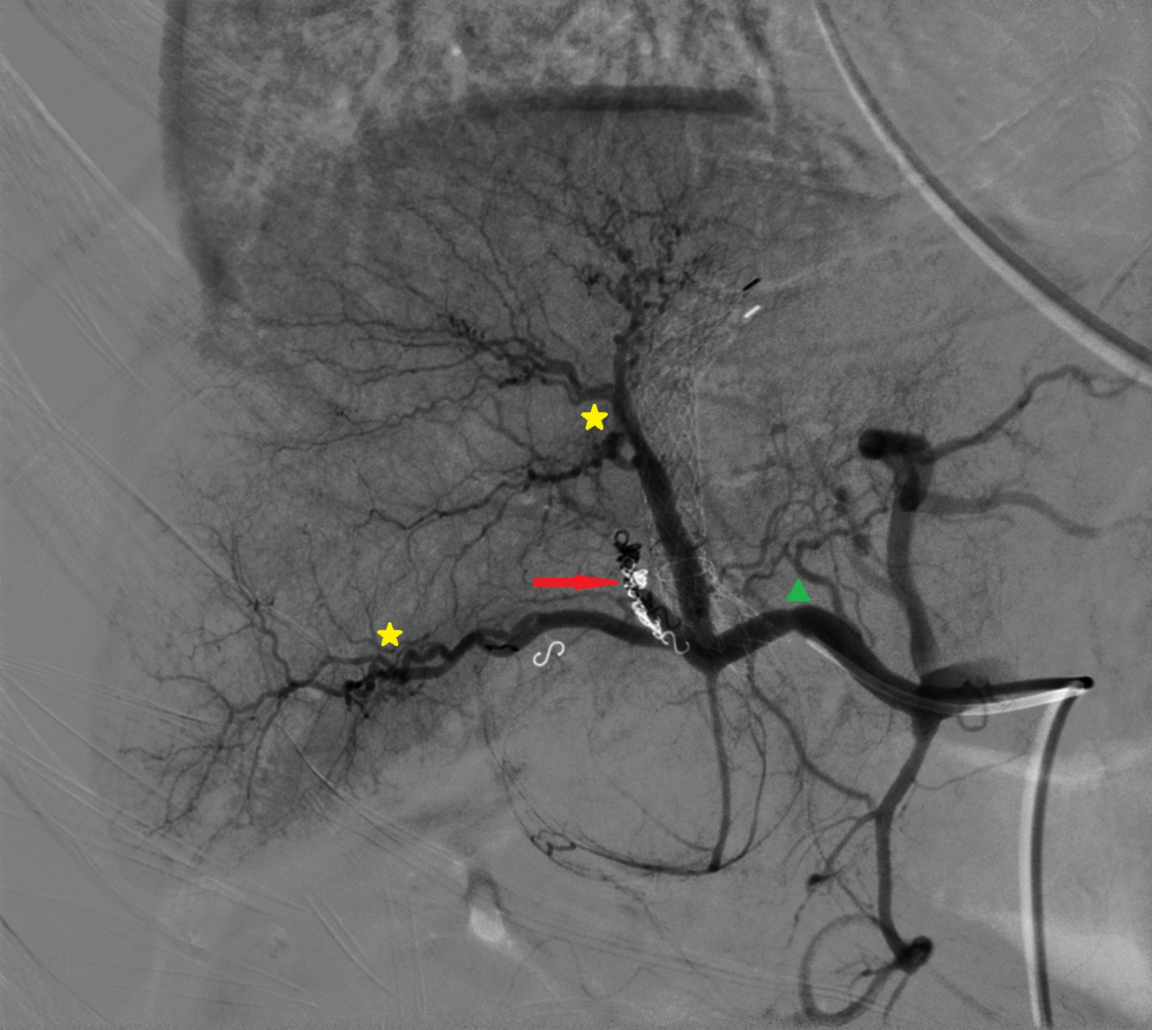
Final hepatic artery angiography Coils inside the fistula (red arrow); patency of the right hepatic artery (green triangle) and its distal branches (yellow stars)

Final angiogram demonstrated the exclusion of the hepatic vein and the biliary tract and the patency of the right hepatic artery and its distal branches (Figure 8).

## Discussion

A transjugular intrahepatic portosystemic shunt (TIPS) has been utilized in the treatment of portal hypertensive complications for decades. Indications for TIPS include management of variceal bleeding, refractory cirrhotic ascites, hepatorenal syndrome, gastric antral vascular ectasia, Budd Chiari syndrome, and refractory hepatic hydrothorax [4].

Iatrogenic hemobilia may occur as a result of hepato-biliary procedures. Given the close proximity of bile duct radicals to the branches of the hepatic artery and portal vein, the incidence of concurrent injury to these structures and consequent fistula formation is not unexpected [5-7]. A 3.8% incidence of hepatic vascular abnormalities was found following percutaneous transhepatic cholangiography, a 5.4% incidence of hepatic vascular abnormalities following percutaneous liver biopsy, and a 26.2% incidence following the placement of indwelling transhepatic drainage catheters [8-9]. The frequency of clinical hemobilia ranges from less than 1% for a liver biopsy to 4% for transhepatic cholangiography, 3% to 14% for percutaneous transhepatic catheter drainage, and <5% post-TIPS procedure [10].

Often hemobilia is a self-limiting phenomenon and expectant observation is a commonly used option. Clinically significant hemobilia presents with biliary colic, jaundice, and gastrointestinal bleeding, which may range from occult to massive bleeding.

The initial diagnosis can be made with endoscopy [11]. CT-A is an accurate, cost-effective tool to assess hemobilia and can show the precise location of bleeding, thereby directing further management [12].

Angiography with embolization is the treatment of choice for most cases of hemobilia [13-14]. The goals of therapy in cases of hemobilia are to stop the bleeding and to restore bile flow. Angiography is clearly the most efficacious method for controlling intrahepatic bleeding sources, with success rates above 95%. A complication of embolization includes hepatobiliary necrosis (6%), abscess formation (9%), bleeding (6%), and gallbladder fibrosis (2%) [15-17].

In this specific case, the bleeding was clinically significant and was a life-threatening event. On the other hand, the therapeutic possibilities were limited by the fact that embolization of the hepatic artery by microparticles or endovascular ligation was highly likely to cause hepatic ischemia also due to the presence of a properly functioning TIPS that stole the portal blood flow. The real therapeutic challenge was to stop the bleeding without interfering with the normal anatomy of the biliary and vascular structures. The only therapeutic possibility was to fill the fistulous tract with spirals without sacrificing arterial branches. The result at the end of the procedure is clearly recognizable by the angiographic images.

## Conclusions

Hemobilia is one of the possible complications of TIPS. A liver parenchymal puncture during a TIPS procedure may damage vascular structures such as the hepatic artery, portal vein, as well as bile duct. In cases of gastrointestinal hemorrhage after TIPS placement, a suspicion of hemobilia should be high. After the diagnosis of hemobilia is confirmed by upper endoscopy or CT-A, transcatheter embolization is the gold standard in the management of hemobilia.
